# Heat-responsive and time-resolved transcriptome and metabolome analyses of *Escherichia coli* uncover thermo-tolerant mechanisms

**DOI:** 10.1038/s41598-020-74606-8

**Published:** 2020-10-19

**Authors:** Sinyeon Kim, Youngshin Kim, Dong Ho Suh, Choong Hwan Lee, Seung Min Yoo, Sang Yup Lee, Sung Ho Yoon

**Affiliations:** 1 Department of Bioscience and Biotechnology, Konkuk University, Seoul, 05029 Republic of Korea; 2 School of Integrative Engineering, Chung-Ang University, Seoul, 06974 Republic of Korea; 3 Metabolic and Biomolecular Engineering National Research Laboratory, Department of Chemical and Biomolecular Engineering (BK21 Plus Program), BioProcess Engineering Research Center, Center for Systems and Synthetic Biotechnology, Institute for the BioCentury, KAIST, Daejeon, 34141 Republic of Korea

**Keywords:** Gene expression profiling, Transcriptomics, Bacterial systems biology

## Abstract

Current understanding of heat shock response has been complicated by the fact that heat stress is inevitably accompanied by changes in specific growth rates and growth stages. In this study, a chemostat culture was successfully performed to avoid the physico-chemical and biological changes that accompany heatshock, which provided a unique opportunity to investigate the full range of cellular responses to thermal stress, ranging from temporary adjustment to phenotypic adaptation at multi-omics levels. Heat-responsive and time-resolved changes in the transcriptome and metabolome of a widely used *E. coli* strain BL21(DE3) were explored in which the temperature was upshifted from 37 to 42 °C. Omics profiles were categorized into early (2 and 10 min), middle (0.5, 1, and 2 h), and late (4, 8, and 40 h) stages of heat stress, each of which reflected the initiation, adaptation, and phenotypic plasticity steps of the stress response. The continued heat stress modulated global gene expression by controlling the expression levels of sigma factors in different time frames, including unexpected downregulation of the second heatshock sigma factor gene (*rpoE*) upon the heat stress. Trehalose, cadaverine, and enterobactin showed increased production to deal with the heat-induced oxidative stress. Genes highly expressed at the late stage were experimentally validated to provide thermotolerance. Intriguingly, a cryptic capsular gene cluster showed considerably high expression level only at the late stage, and its expression was essential for cell growth at high temperature. Granule-forming and elongated cells were observed at the late stage, which was morphological plasticity occurred as a result of acclimation to the continued heat stress. Whole process of thermal adaptation along with the genetic and metabolic changes at fine temporal resolution will contribute to far-reaching comprehension of the heat shock response. Further, the identified thermotolerant genes will be useful to rationally engineer thermotolerant microorganisms.

## Introduction

Microorganisms respond to environmental changes by invoking various stress responses^1^. The heat shock response (HSR) is a primary defense and protection mechanism and is generally regarded as the cellular stress response to a sudden temperature increase, which leads to a transient over-synthesis of heat shock proteins (HSPs)^2–5^. The primary function of HSPs is to maintain protein-folding homeostasis. In this process, chaperones promote protein folding and proteases degrade misfolded or aggregated proteins. In *Escherichia coli*, the synthesis of major HSPs is triggered by a transient increase in *σ*^H^, a major heatshock sigma factor encoded by *rpoH*^4^.

Most HSR studies have been performed in batch (flask) cultures by imposing a short temperature upshift on various organisms^6–11^. In such experimental setups, the HSR is inevitably accompanied by changes in specific growth rates and growth stages. Thus, whether these observed expression patterns can be exclusively attributed to heatshock is unclear^12^. Moreover, the medium composition and accumulation of byproducts, including acetate and lactate, keep changing during batch cultivation, which consequently complicates the interpretation of these experiments.

Steady-state continuous cultivation (i.e., chemostat) is a highly reproducible experimental approach to precisely control the growth rate and culture condition, and thus has proven ideal for the omics analyses of various environmental stresses^13,14^. However, there have been only a few attempts to use chemostats for HSR studies. At the transcript level, RT-PCR analysis was conducted for a small number of genes from cells reaching a new steady state after temperature upshift^15^. At the protein level, two-dimensional gel electrophoresis was used to identify temperature-induced proteins by comparing *E. coli* growing exponentially (37 °C) with those at stationary phase (47.5 °C) in a cascade of two continuously operated bioreactors^16^. However, these HSR studies compared two samples at normal and heatshock temperatures and thus provided only a generalized snapshot of a genome-wide response to thermal stress.

Beyond the aspect of fundamental biology, the understanding of the HSR mechanism has important industrial applications, such as the production of thermo-induced recombinant proteins^17^ as well as simultaneous saccharification and fermentation (SSF) for biofuel production^18^. Moreover, as the heat produced during industrial high cell density cultures can heavily reduce the productivity of biomass and recombinant proteins, the development of heat-tolerant host strains has long been a research priority^19^.

*E. coli* B strains, especially BL21(DE3), have been widely used as hosts for the overproduction of recombinant proteins, ethanol, and other biomolecules^20,21^. In this study, we analyzed the dynamic transcriptomic and metabolomic changes in *E. coli* BL21(DE3) exposed to continued heat stress. A chemostat culture was run over 40 h after the culture temperature was suddenly raised from 37 to 42 °C. The time-series samples were subjected to transcriptome and metabolome analyses using whole genome high-resolution tiling arrays and gas chromatography‐time of flight-mass spectrometry (GC‐TOF‐MS), respectively. Interpretations from the omics analyses included reactive oxygen species (ROS) measurement, morphology inspection, and experimental validation.

## Results and discussion

### Chemostat culture of *E. coli* exposed to continued heatshock

**Figure 1:**
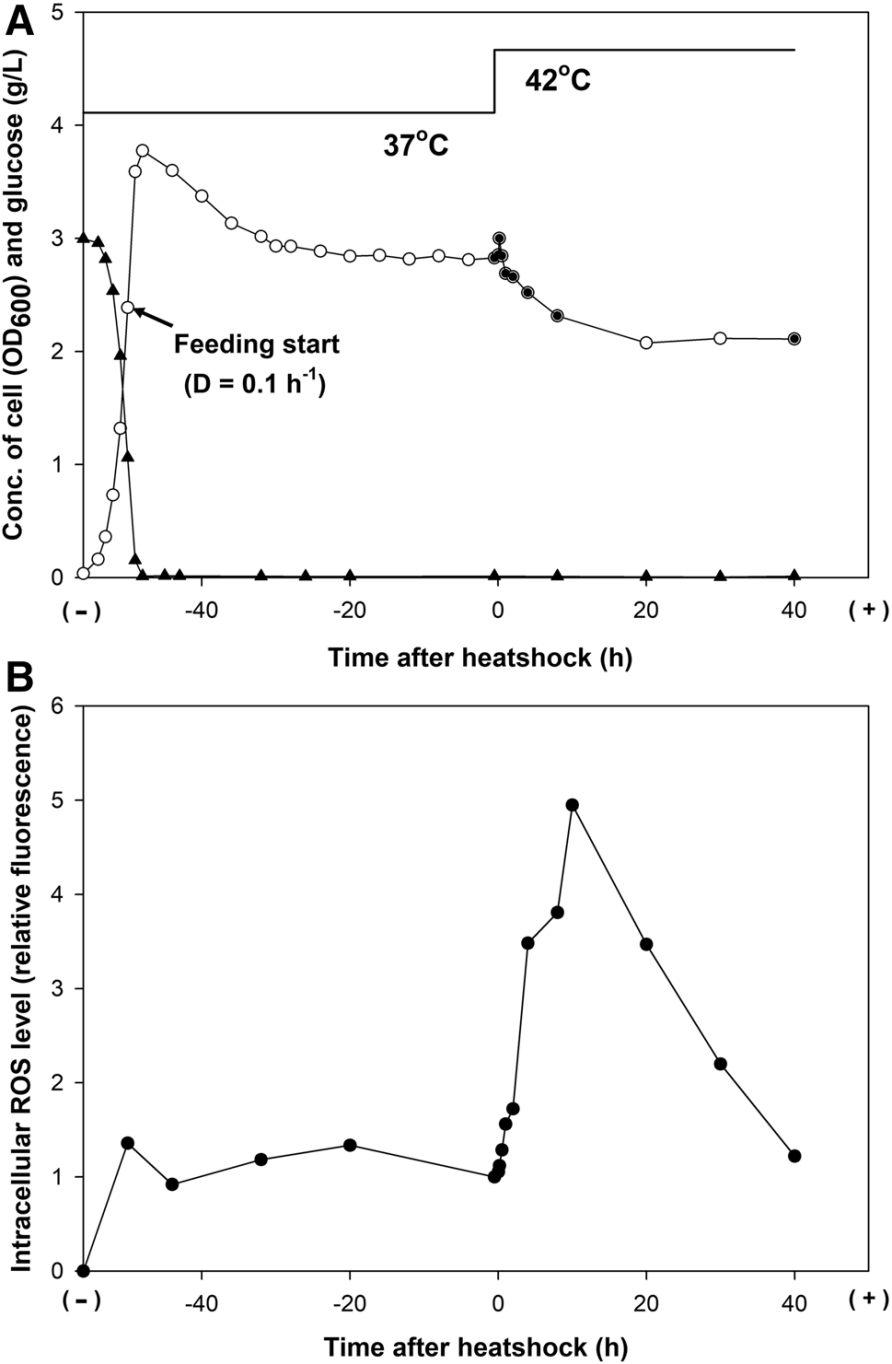
Chemostat culture of *E. coli* BL21(DE3) under heatshock stress. (**A**) Time profiles of the cell density (open circle) and glucose concentration in the culture media (filled triangle). The filled circles indicate the sampling points for transcriptome and proteome analyses, which were done 30 min before the temperature increase (from 37 to 42 °C) and after 2 min, 10 min, 30 min, 1 h, 2 h, 4 h, 8 h, and 40 h. (**B**) Time profiles of the intracellular reactive oxygen species (ROS) level.

A chemostat culture was performed in glucose-limited defined medium to maintain the growth rate of the cells and constant culture conditions that included pH, dissolved oxygen (DO), and medium composition (Fig. 1). During the continuous mode, a dilution rate of 0.1 h^−1^ was chosen because it allowed stable exponential growth without the accumulation of glucose and byproducts, such as acetate and lactate, in the culture medium^22^; moreover, it corresponded to a mean residence time of 10 h (i.e., the cells were incubated for an average time of 10 h). The chosen conditions allowed a detailed investigation of the time-resolved cellular responses during the bacterial lifetime.

After the initial batch mode, cells were grown for more than five residence times to reach a steady state at 37 °C. Upon the temperature shift to 42 °C, the cell density, determined by the optical density at 600 nm (OD_600_), transiently increased from 2.8 to 3.0 in 10 min. Then, the cell density decreased gradually to 2.1 in 20 h and remained almost the same until the end of the chemostat run. Residual glucose and the accumulation of acetate, lactate, and formate were hardly detectable in the culture medium. The ROS level increased up to approximately five-fold in 10 h after the temperature upshift to 42 °C and decreased by the same level at 37 °C.

### Transcriptomic response to heat stress

Using single-colored tiling arrays, mRNA abundances were measured for the nine time-series samples (one at 37 °C and eight at 42 °C) (Fig. 1). Among 4439 feature loci of the BL21(DE3) genome^23^, 3413 loci were expressed in at least one of the nine time-series array data (*p* value < 0.01) (Supplementary Table S1). The changes in mRNAs at eight time points at 42 °C were calculated with reference to an RNA sample from the steady-state culture at 37 °C. Genes showing the expression levels of ≥ 2- or ≤ 0.5-fold were considered differentially expressed genes (DEGs) and were identified at each time point (Supplementary Fig. S1). The functions of the up and downregulated DEGs were categorized by clusters of orthologous groups (COGs)^24^. The number of DEGs tended to decrease with the time of incubation at 42 °C, from 455 genes at 2 min to 289 genes at 40 h. During the initial 10 min, the downregulated genes outnumbered the upregulated genes by two-fold. The difference diminished toward the end of the culture. There was a poor overlap among the DEGs at each time point, and only 55 upregulated and 106 downregulated genes were identified as DEGs throughout the culture. This indicates highly dynamic transcriptome changes during the continued heatshock.

Cellular processes in response to environmental perturbations are transcriptionally coordinated in magnitude and time^25,26^. To obtain insight into transcriptome dynamics, we identified genes that were differentially expressed over time. To this end, we used Transcript Time Course Analysis (TTCA)^27^ which is designed to analyze time-series microarray data from perturbation experiments to discriminate the early and late changes in gene expression. Specifically, TTCA is intended for experimental designs with sparse and irregularly sampled time course gene expression data sets^27^. TTCA calculates the integral scores quantifying the absolute expression changes in different time intervals, considering the inherent ordering and spacing information provided by the time points. We considered three time intervals which were identified by hierarchical clustering and principal component analysis (PCA) showing that the eight time points were clustered into three time periods: early (2 and 10 min), middle (0.5, 1, and 2 h), and late (4, 8, and 40 h) period of the upshifted temperature (Supplementary Fig. S2).

**Figure 2:**
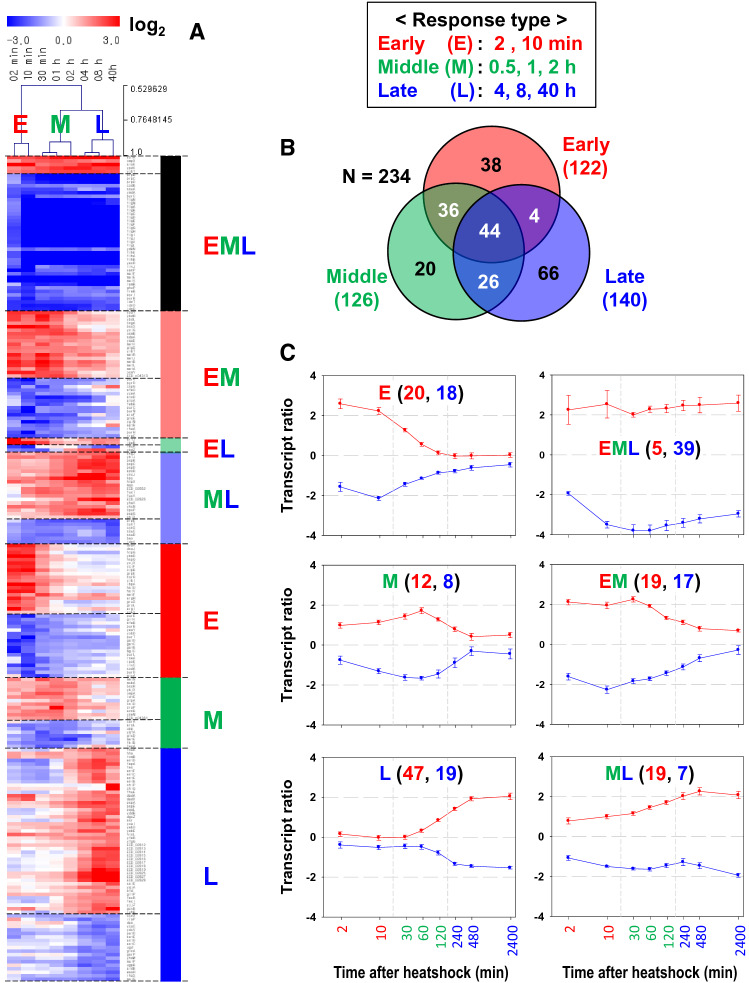
Genes differentially expressed at the early, middle, and late periods of the upshifted temperature. The mRNA log_2_ ratios of time-series data were normalized to a sample from pre-perturbation (30 min before the temperature upshift). (**A**) Heatmap of the transcript ratios grouped according to time stages with member genes in rows and sampling time points in columns. Clustering of the sampling time points is shown above the heat map for the early (E), middle (M), and late (L) stages. The image was created with MeV software (version 4.9.0, https://mev.tm4.org). (**B**) Venn diagram of the differentially expressed genes (DEGs) identified in the different time stages. (**C**) Time profiles of averaged transcript ratios of the DEGs according to the time stage. The X-axis denotes the culture time after the temperature upshift (min in log scale), and the Y axis denotes the log_2_-transformed transcript ratio in reference to the mRNA intensity at 30 min before the perturbation. The error bar denotes the standard error of the mean from the member genes. In each plot, the numbers of upregulated and downregulated genes are depicted in red and blue, respectively.

Three separate integral scores of the early, middle, and late stages were computed for 3413 loci that expressed over the nine time-series data, and genes showing significant dynamics (*p* value < 0.05) were identified from the log-normal distribution function providing the best fit of the distribution of the integral score values at each time interval (Supplementary Fig. S3). A total of 234 genes were significant in at least one of the three integral scores of the time intervals and 44 genes were significant in all stages. (Fig. 2 and Supplementary Table S2). The numbers of genes showing significant dynamics in the integral scores of the early, middle, and late stages were 122, 126, and 140, respectively. They included stage-specific genes whose transcription was induced or repressed only during one time stage: 38 genes during the early stage, 20 during the middle stage, and 66 during the late stage. Among them, functionally distinct genes were summarized in Table 1 and Supplementary Fig. S4, and described in detail in Supplementary Text. Briefly, early-responsive genes displaying sharp and transient expression changes upon the temperature upshift were mostly associated with the well-known HSPs of chaperones (*dnaKJ*, *grpE*, *ibpA*, and *groESL*) and proteases (*htpG*, *hspQ*, *clpB*, and *hslUV*) for protein folding and degradation, respectively, in the cytoplasm. Interestingly, four genes with unknown functions (*ybeD*, *ycjX*, *ycjF*, and *yibI*) were identified as early-responsive genes. Genes highly expressed only during the late stage were associated with enterobactin biosynthesis (*entCEB*, *entD*, and *fes*-*entF*) and capsular polysaccharide biosynthesis encoded by the *kps* gene cluster (ECD_02813 to ECD_02828).

**Table 1:** Functional enrichment of DEGs characteristic of early, middle, and late responses.

Class^a^	Gene Ontology (GO) function	False discovery rate	Genes
E.high	Response to heat (GO:0009408)	9.71E−13	*clpB*, *dnaKJ*, *groSL*, *grpE*, *hslVU*, *hspQ*, *htpG*, *ibpA*
E.low	Purine biosynthesis (GO: 0009127)	1.83E−04	*purB*, *purD*, *purE*, *purL*, *purT*
M.high	Not detected	–	*–*
M.low	Not detected	–	*–*
L.high	Enterobactin metabolic process (GO:0009238)	4.75E−06	*entCEB*, *entD*, *fes-entF*
L.low	Not detected	–	–
EML.high	Not detected	–	*–*
EML.low	Flagellum organization (GO:0044781)	1.89E−09	*flgAMN*, *flgD*, *flgGJK*, *flhBAE*
	Disaccharide transport (GO:0015766)	1.77E−04	*malK-lamB*, *malEF*, *treB*
EM.high	Methionine biosynthesis (GO:0009086)	3.60E−03	*metA*, *metBL*, *metJ*, *metR*
EM.low	Biosynthesis of purine and pyrimidine (GO:0009156)	2.28E−03	*purC*, *purH*, *purK*, *purM*, *pyrD*
ML.high	Phage shock (GO:0009271)	6.34E−03	*pspBD*, *pspG*
ML.low	Not detected	–	–

^a^Time stages are early (E; 2 and 10 min), middle (M; 0.5, 1, and 2 h), and late (L; 4, 8, and 40 h), and the gene expression is high or low. See Fig. 2C for the gene expression pattern.

Global gene expression was coordinated by the expression levels of sigma factors in different time frames (Supplementary Fig. S5). The major heat-inducible sigma gene (*rpoH*) and the predominant vegetative sigma gene (*rpoD*) were highly expressed upon the temperature upshift. Expression level of the stationary phase sigma factor gene (*rpoS*) did not respond appreciably to the temperature upshift, indicating that the heatshock condition in this study was not accompanied by entry into the stationary phase. Unexpectedly, the second heatshock sigma factor gene (*rpoE*) was downregulated within 10 min after the heat stress and gradually returned to the level in unstressed cells. The dynamic expression changes of the sigma factor genes are described in detail in Supplementary Text and Supplementary Fig. S6.

### Metabolomic response to heat stress

GC–TOF–MS analysis was used to assess the changes in the intracellular metabolite profiles in response to heat stress. GC–TOF–MS detected 45,489 variables in all samples, which were then subjected to principal component analysis (PCA) (Supplementary Fig. S7). Consistent with the clustering of the transcriptome data, the partial least squares-discriminant analysis (PLS-DA) score plot derived from the value of the PCA model showed the clear separation of the three stages: the early (2 and 10 min), middle (0.5, 1, and 2 h), and late (4, 8, and 40 h) responses (R2X = 0.355, R2Y = 0.987, Q2 = 0.836, *p* value < 0.05).

**Figure 3:**
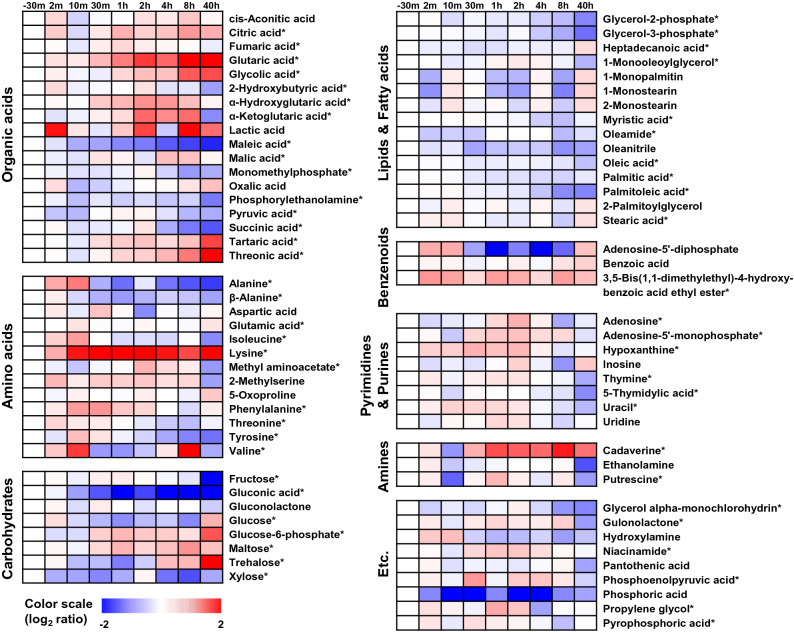
Time profiles of the intracellular metabolites under heat stress. Seventy-seven metabolites were identified via the GC-TOF–MS analysis and are listed in Supplementary Table S3. Fifty-seven of these metabolites that were significantly differentially expressed among the samples (VIP > 1.0 and *p* < 0.05) are indicated by asterisks. Fold-change was calculated as the relative abundance of each metabolite in every sample relative to that in the sample taken before the perturbation. The metabolic alternations are depicted as heatmaps on a log2 scale.

Variable importance in projection (VIP) scores rank the overall contribution of each variable to the PLS-DA model. Variables with VIP > 1.0 are considered statistically significant in this model. In this study, VIP analysis was initially applied to obtain the significant variables that could be used for metabolic pathway analysis. Of a total of 77 metabolites identified, the levels of 57 metabolites were significantly altered among the samples based on their VIP values (> 1.0) from the PLS‐DA models and *p* values (< 0.05) from one‐way ANOVA (Fig. 3 and Supplementary Table S3). These 57 metabolites comprised organic acids (n = 15), amino acids (n = 10), lipids (n = 10), carbohydrates (n = 7), nitrogenous bases (n = 6), amines (n = 2), a benzenoid (n = 1), and miscellaneous metabolites (n = 6). Metabolites that varied significantly among the time stages were visualized by heat map analysis (Fig. 3). The metabolites selected displayed greater than ± 1.0 of log_2_ scale fold-changes normalized by the sample (negative control) from the steady-state culture at 37 °C. Following the shift to 42 °C, there was pronounced accumulation of glutarate, cadaverine, trehalose, threonate, and especially lysine. The accumulation of phosphoric acid, gluconic acid, maleate, xylose, alanine, valine, and adenosine-5′-diphosphate was low, except for the transient high accumulation of alanine and adenosine-5′-diphosphate in the first 10 min after the temperature upshift.

### Morphological response to heat stress

**Figure 4:**
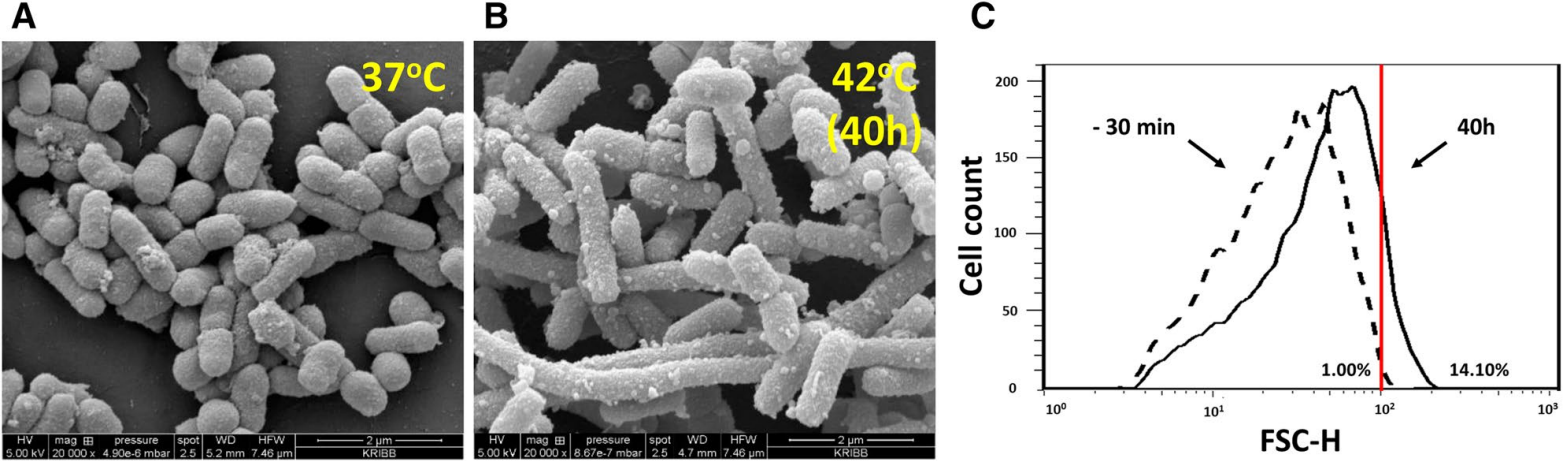
Morphological changes of *E. coli* during heatshock. Scanning electron micrographs of bacterial cells grown in the chemostat culture at 37 °C 30 min before the temperature upshift (− 30 min sample) (**A**) and 40 h after the temperature upshift to 42 °C (**B**). Cell size distributions determined using flow cytometry analysis (**C**). Dotted and solid lines denote cells at 37 °C 30 min before the temperature upshift and 40 h after the shift to 42 °C, respectively. The vertical red line represents the longest 1% size of the -30 min sample, which was used as the threshold. The mean forward scatter height (FSC-H) on the X axis represents the length of the rod-shaped bacteria. Percentages of elongated cell size in the total cells are 1% for the − 30 min samples and 14.10% for 40 h samples.

As environmental changes often influence bacterial shape^28^, we monitored cell morphology using flow cytometry and SEM throughout the culture (Fig. 4). Cell size distribution was analyzed based on forward scatter (FSC) in flow cytometry (Supplementary Fig. S8). FSC correlates mainly with the longest diameter of rod-shaped bacteria, and it has been reported to agree well with microscopic observations^29,30^. FSC corresponding to the largest 1% in the size distribution of the cells at steady-state growth at 37 °C was used as the threshold to estimate the fraction of large-sized cells at each of the sampling time points. After the temperature upshift to 42 °C, the mean cell size and the proportion of large-sized cells kept increasing with culture time. The large-sized cell fraction comprised 14.1% of the cells cultured for over 40 h at 42 °C. To visually inspect the morphological change, cells cultured at 37 °C (30 min before the temperature upshift) and 42 °C (40 h after the temperature upshift) were examined by SEM. The images showed that the heat-shocked cells were elongated or even filamentous. They also displayed granule-like appendages on the cell surface.

To discern whether the morphological change resulted from genetic mutation or nonheritable phenotypic plasticity, we performed microbial experimental evolution^31^. Chemostat culture samples 40 h after the temperature upshift were propagated at 37 or 42 °C in MR medium by transferring 0.25 mL of culture into 25 mL of fresh medium every 12 h. After 16 serial transfers (equivalent to about 100 generations), samples were examined by SEM (Supplementary Fig. S9). The images revealed that the filamentous, granule-forming *E. coli* returned to their regular size and shape after experimental evolution at 37 °C and 42 °C. These findings indicated that the morphological changes were a result of the acclimation to heat stress through phenotypic plasticity and not due to a completely novel phenotype acquired through genetic mutation.

### Experimental verification of thermotolerant genes

**Figure 5:**
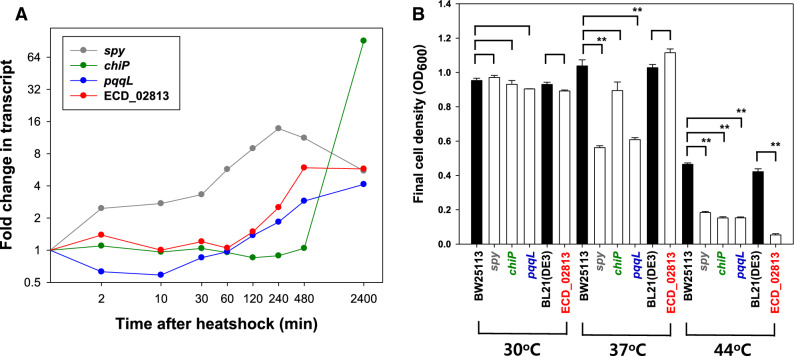
Effect of heatshock-responsive gene (*spy*, *chiP*, *pqqL*, and ECD_02813) deletion on bacterial growth with temperature. (**A**) Temporal expression levels. The X-axis denotes the culture time after the temperature upshift (min in log scale). The Y axis denotes the log_2_-transformed transcript ratio in reference to the mRNA intensity 30 min before the temperature upshift. (**B**) Final cell densities of the deleted mutant strains (in white bars) and their background strains (black bar) grown in MR medium for 24 h at 30 °C, 37 °C, and 44 °C (**p < 0.01). The error bars represent the standard deviation of the mean from three independent cultivations.

Since some genes were highly expressed at the end of the culture, we tested the effect of the knockout of these genes. We focused on genes encoding ATP-independent periplasmic chaperone (*spy*), outer membrane porin for chitooligosaccharides (*chiP*, formerly *ybfM*), putative periplasmic zinc metallopeptidase (*pqqL*), and a hypothetical protein (ECD_02813) (Fig. 5A). The *spy* gene was chosen because of its consistent high activation throughout the temperature upshift, in contrast to an impulse-like expression pattern of most other chaperones. *chiP* was highly activated only at the end of the perturbation, as was ECD_02813 (the first gene of the biosynthetic gene cluster of capsular polysaccharides). *pqqL* was downregulated during the early stage and gradually upregulated thereafter. Compared with the wild type (WT), all the gene deletion mutants showed similar cell growth at 30 °C (Fig. 5B and Supplementary Fig. S10A). At 37 °C, the deletion mutants, except ΔECD_02813, displayed slower cell growth with a lower final cell density. However, at 44 °C which was the highest temperature allowing *E. coli* growth using the MR defined medium in our preliminary experiment, the growth of all the mutants was significantly reduced. The temperature-dependent growth might indicate that *spy*, *chiP*, *pqqL*, and ECD_02813 play a role in thermotolerance.

Intrigued by the lack of growth of ΔECD_02813 at 44 °C, we constructed a complementation system expressing the plasmid-encoded gene cluster of ECD_02813-02819 in ΔECD_02813, since ECD_02813 was the first gene of the seven-gene operon (Supplementary Fig. S4D). The expression of ECD_02813-02819 in the WT strain did not improve cell growth at 37 °C, however, did restore the cell growth to a level comparable to that of the WT strain grown at 44 °C (Supplementary Fig. S10B). The gene deletion and complementation results strongly suggested that the expression of the biosynthetic gene cluster of capsular polysaccharides contributes to improved cell growth at high temperatures.

### Metabolic adaptation to heat stress

Microorganisms often respond to environmental stresses by diverting cellular resources from biomass synthesis to the restoration of homeostasis^25^. In our study, the expression levels of metabolic genes were continually readjusted during the continued heat stress (Supplementary Fig. S11). Tricarboxylic acid (TCA) cycle genes were downregulated by approximately 0.7-fold within 10 min after the temperature upshift and gradually increased thereafter. Particularly, the expression of genes associated with two consecutive steps of the TCA cycle responsible for the conversion of malate to oxaloacetate by malate dehydrogenase (encoded by *mdh* and *mqo*) and then to citrate by citrate synthase (*gltA*) kept increasing up to approximately 1.4-fold. This agreed with a previous *E. coli* proteome analyses of the HSR, which revealed that malate dehydrogenase was highly expressed under heatshock conditions, with the solubility of the recombinant proteins being dramatically increased when expressed with malate dehydrogenase as a fusion expression partner^32^. Another *E. coli* proteome analysis observed the high expression of citrate synthase under heat stress^16^.

Operons for cytochrome *bo*_*3*_ terminal oxidase (*cyoABCDE*) and ATP synthase (*atpIBEFHAGDC*) were also downregulated (by approximately 0.5-fold) upon heat stress and were restored to their levels before perturbation. Metabolome analysis also showed that the phosphate (detected as phosphoric acid) required for ATP synthesis was decreased upon the temperature increase. The transiently decreased expression of genes involved in the TCA cycle and ATP synthesis suggested that thermal stress can trigger the reduced activity of aerobic respiration upon heat stress, which recovers when the cells adapt to the stress.

Genes involved in the biosynthesis pathways of purine and pyrimidine nucleotides (*purA*, *purB*, *purC*, *purEK*, *purF*, *purHD*, *purl*, *purMN*, *purT*, *pyrBI*, *pyrC*, *pyrD*, *pyrE*, *pyrF*, *pyrG*, *ndk*, *guaBA*, and *pyrH*) were downregulated at 42 °C, especially within 10 min after the perturbation (≤ 0.5-fold). Most of the genes involved in amino acid biosynthesis pathways did not vary appreciably at 42 °C, except for those involved in the biosynthesis pathways of histidine (*hisGDCBHAFI*) and arginine (*argCBH*, *argA*, *argD*, *argE*, *argG*, and *argI*), which were highly expressed, especially during the initial 10 min. Genes involved in methionine metabolism exhibited complicated expression patterns upon heat stress. The genes involved in the conversion of aspartate to homoserine (*metL*) and then to homocysteine (*metA*, *metB*, and *metC*) as well as the *metK* gene required for the conversion of methionine to S-adenosyl-L-methionine showed continued high expression (approximately fourfold), especially within the initial 10 min after the temperature upshift. In contrast, genes necessary for the conversion of homocysteine to methionine (*metE* and *metH*) were downregulated by approximately 0.5-fold. When *E. coli* growing in a defined medium are exposed to elevated temperatures, the intracellular methionine concentration becomes limited and cell growth stops^33^. Methionine deficiency is mainly caused by the thermally unstable *metA* gene product, homoserine trans-succinylase, which catalyzes the first step in the methionine biosynthesis pathway and is induced by elevated temperatures^34^. While most amino acid biosynthetic genes are organized into one operon, methionine biosynthetic genes are scattered throughout the *E. coli* genome^35^. The expression of *met* genes is regulated by multiple promoters mediated by the MetJ repressor and the MetR activator. While *metJ* and *metR* have conflicting roles in the regulation of the *met* genes, in our study, both were induced during the early and middle stages (Supplementary Table S1). This might suggest the existence of as-yet unknown regulatory mechanisms for methionine biosynthesis.

### Protectants against heat-induced oxidative stress

Heat stress can generate large amounts of ROS, which include superoxide anions and hydrogen peroxide, resulting in oxidative stress^36^. To scavenge ROS, cells need to produce antioxidants. The induction of oxidative stress has been reported in previous experiments in which rapidly growing *E. coli* exposed to heatshock entered the stationary phase^16,37–39^. In our study, a temperature increase from 37 to 42 °C in the aerobic *E. coli* chemostat resulted in the generation of up to fivefold more ROS in 10 h, which was finally fully restored to the level at 37 °C (Fig. 1). The *sodC* gene encoding periplasmic superoxide dismutase was highly expressed. However, the *sodA*, *ahpC*, and *katE* antioxidant genes were not. Interestingly, genes involved in iron metabolism, which included enterobactin biosynthesis (*entCEB*, *entD*, and *fes*-*entF*), iron uptake (*fhuA* and *fhuE*), regulator of *fec* operon (*fecR*), sRNA involved in iron homeostasis (*ryhB*), bacterioferritin-associated ferredoxin (*bfd*), and iron-starvation sigma factor (*fecI*), began to be upregulated 2 h after the temperature increase. The increases were profound by the end of the culture. Enterobactin is a siderophore that has cytoprotective activity against oxidative stress. The transcription and production of enterobactin are induced in *E. coli* exposed to either hydrogen peroxide or paraquat, even under conditions of excess iron^40^.

Heat-inducible metabolites are essential for protecting cells from oxidative stress. Our metabolome analysis showed that significant accumulation of lysine at 42 °C preceded the incremental increase of cadaverine and glutarate (Fig. 3), indicating the activation of the metabolic pathway from lysine via cadaverine to glutarate. The *ldcC* gene, which encodes lysine decarboxylase for the conversion of L-lysine into cadaverine, was upregulated. Cadaverine is a cellular polyamine that has an important protective role under conditions of acidic pH, oxidative stress, and anaerobic phosphate (Pi) starvation^41,42^. SodA induction was considerably reduced by the increased formation of cadaverine in *Vibrio vulnificus*, demonstrating that cadaverine can function as an ROS scavenger^43^. Cadaverine is also associated with the biosynthesis of siderophores^44^.

Among the carbohydrates identified from the metabolome analysis, trehalose accumulation began 4 h after the temperature increase and gradually reached its maximum (sevenfold) by the end of the culture period (Fig. 3). The disaccharide trehalose prevents proteins from denaturation and aggregation, and its protective role in heatshock has been extensively studied in yeast^45^. Trehalose is biosynthesized from UDP-glucose and glucose-6-phosphate, and it can be degraded into two molecules of glucose that enter the glycolytic pathway. Genes for trehalose biosynthesis (*otsBA*) and cytoplasmic trehalase (*treF*) were transiently induced upon the temperature shift. The different accumulation patterns of internal trehalose and cadaverine might suggest that these molecules have distinct protective roles.

### Morphological plasticity by acclimation to continued heat stress

Many bacteria produce capsular polysaccharide (K) antigens on their surfaces to protect themselves from various environmental stresses^46,47^. Extraintestinal pathogenic isolates of *E. coli* usually express group II capsules^48^. Distinct from other groups, group II capsules are thermo-regulated and are not expressed below 20 °C^48^, which ensures that capsule expression is turned on inside the host but not in the external environment. Group 2 K1 capsule biosynthesis genes of *E. coli* are organized in three regions^49^. The region 1 genes encode enzymes for the export and translocation of mature polysaccharides, and the region 3 genes encode the ATP-binding-cassette transporter. Region 2 gene products are involved in the biosynthesis and polymerization of the K1 polysaccharide. In the group II capsular gene cluster of *E. coli* BL21(DE3), region 1 and 3 genes are functional. However, region 2 contains insertion sequence (IS) elements, which render this strain non-encapsulated^50^. Unexpectedly, the SEM images revealed granule-like appendages on the surface of the elongated or filamentous cells grown at 42 °C for 40 h (Fig. 4). The appendages are not likely to be colanic acid (CA) as the whole gene cluster for CA biosynthesis was hardly expressed at 37 °C and 42 °C. In addition, CA production is decreased at high temperature and is undetectable at 42 °C^51^. Presumably, the IS insertion at region 2 in BL21(DE3) did not completely abolish the sugar production, and the non-polymerized sugar was presented on the cell surface.

When the granule-forming and elongated cells were serially transferred in shake flask cultures at either 37 °C and 42 °C, the morphology returned to the regular rod-like form (Supplementary Fig. S9). Organisms acclimate to environmental stresses via two phases of adaptation involving physiological acclimation by phenotypic plasticity in the short-term and adaptive evolution by genetic changes in the long-term^52^. To cope with a sudden change in environmental conditions, cells can rewire their transcriptional regulatory network by controlling both the expression levels of the responding genes and the timing of the response^26,53^. Long-term experimental evolution studies with microorganisms have been widely performed to correlate genetic mutations to phenotypic changes by comparing ancestral and evolved strains^54–56^. However, the mechanism underlying the physiological acclimation to thermal stress remains largely unexplored. Our findings indicate that the morphological change was a result of acclimation to the continued heat stress through phenotypic plasticity, with the expression of genes producing thermotolerance.

### Identification of genes conferring thermotolerance

The heat-responsive profiles of the transcriptome and metabolome can be classified into early (2 and 10 min), middle (0.5, 1, and 2 h), and late (4, 8, and 40 h) stages (Fig. 2). The poor overlap among DEGs at each time point suggested the distinct physiological role of each stage. At the early stage, a damage control response is invoked, such as the involvement of protein chaperones. This response is a prompt and transient event, which is widespread in environmental stresses and is related to cross-protection^2^. When the heat stress persisted, gene expression level can settle at a new steady state, and genes that are highly expressed at the late stage could be potential targets for conferring thermotolerance. To test this hypothesis, single-deletion mutants of the relevant genes (*spy*, *chiP*, *pqqL*, and ECD_02813) were constructed. All showed growth defects at 44 °C, but not at 30 °C and 37 °C. Particularly, the deletion and complementation of region 1 of the capsular gene cluster (ΔECD_02813) suggest that the expression of the capsule gene cluster is beneficial only for cell growth at severe heatshock conditions of 44 °C (Fig. 5B and Supplementary Fig. S10). These findings demonstrate that genes that are specifically highly expressed in the late stage of heat stress could be good candidates to confer heat-specific protection.

**Figure 6:**
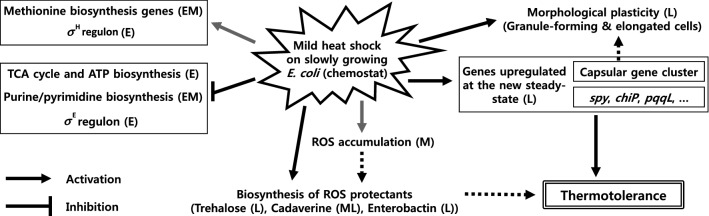
A proposed regulation model of *E. coli* in response to the prolonged heat stress. The solid black lines represent the findings of this study. The solid gray lines denote the present results that agree with those of previous studies. The dotted lines represent the hypothetical links suggested in this study, requiring experimental validation. Arrows represent the activation influences, and blunt-headed lines denote repression. Rectangles depict the biological processes or their associated genes, and the parentheses denote the early (2 and 10 min; E), middle (0.5, 1, and 2 h; M), and late (4, 8, and 40 h; L) stages of heat stress.

Conclusively, results of the multi-omics analysis combined with physiological observations can be distilled into a regulation model of *E. coli* in response to the prolonged thermal stress (Fig. 6). Further, insights and omics data generated in this study expand our knowledge of the HSR and should be useful to rationally engineer thermotolerance in microorganisms.

## Methods

### Bacterial strains and chemostat conditions

The *E. coli* strains and plasmids used in this study are listed in Supplementary Table S4. *E. coli* strain BL21(DE3) was provided by F William Studier, Brookhaven National Laboratory^57^. Modified R (MR) defined medium was used for the seed culture, initial batch culture, and feeding solution. The MR medium (pH 7.0) contained 3 g/L glucose, 4 g/L (NH_4_)_2_HPO_4_, 6.67 g/L KH_2_PO_4_, 0.8 g/L citric acid, 0.8 g/L MgSO_4_·7H_2_O, and 5 mL/L trace metal solution. The trace metal solution contained 0.5 M HCl, 10 g/L FeSO_4_·7H_2_O, 2.2 g/L ZnSO_4_·7H_2_O, 1 g/L CuSO_4_·5H_2_O, 0.5 g/L MnSO_4_·4H_2_O, 0.02 g/L Na_2_B_4_O_7_·10H_2_O, 2 g/L CaCl_2_, and 0.1 g/L (NH_4_)_6_MO_7_O_24_·4H_2_O^23^. Throughout the culture, the pH was maintained at 7.0 by the automatic feeding of 3 M NaOH. The dissolved oxygen concentration was kept above 40% air saturation by supplying air (1.5 L/min) and automatically varying the agitation speed above 300 rpm.

The seed cultures were prepared by growing cells in 125-mL flasks containing 25 mL of MR medium at 37 °C and 200 rpm for 12 h. Next, 10 mL of seed culture was transferred to a 2.5-L BioFlo 310 fermenter (New Brunswick Scientific, Edison, NJ, USA) containing 1 L of MR medium. Cultures were first run in the batch mode at 37 °C. When the cell density (OD_600_) reached 2.4 at the exponential phase, the culture mode was changed to continuous at a dilution rate (D) of 0.1 h^−1^ by the continuous supply of the feeding solution. After the culture reached steady-state (approximately 50 h after the feeding), the temperature was shifted to 42 °C within 3.5 min. The concentrations of glucose, acetate, citrate, formate, and succinate in the culture media were measured using a model 1260 Infinity HPLC device (Agilent Technologies, Santa Clara, CA, USA) equipped with an Aminex HPX-87H ion exchange column (300 × 7.8 mm; Bio-Rad Laboratories, Hercules, CA, USA). For transcriptome and metabolome analyses, samples were taken at nine time points: 30 min before the perturbation and 2, 10, 30, 60, 120, 240, 480, and 2400 min after the start of perturbation.

### Transcriptome analysis

A whole genome high-resolution tiling array was constructed to contain 957515 60-mer probes with strand-specific sequences, in addition to the controls included by the manufacturer (Agilent custom GE microarray, 1 × 1 M)^23^. Probes were tiled every 10 bp (i.e., a 50 bp overlap between adjacent probes) for the BL21(DE3) genome^58^.

Total RNA was prepared using the RNAprotect Bacteria Reagent (Qiagen, Düsseldorf, Germany) and the mirVana miRNA Isolation Kit (Thermo Fisher Scientific Inc., Waltham, MA, USA) as previously described^23^. The purified RNA was directly labeled with Cy3 using a Label IT µArray Labeling Kit (Mirus, Madison, WI, USA) and was hybridized with the tiling array. The array was scanned using a DNA microarray scanner (Agilent Technologies). Signal intensity and local background were determined using Feature Extraction software (Agilent Technologies). Probe intensities from the array experiments were quantile-normalized.

The Transcription Detector algorithm^59^ was used to determine probes that were expressed above the background level with a FDR of 0.01 using the custom-designed 10,000 negative control probes^23^. The statistical significance of the expression of each locus was evaluated by calculating the *p* value estimating the likelihood of overrepresentation of the expressed probes for each locus based on the cumulative hypergeometric distribution. These *p* values were corrected for multiple comparison testing by the Bonferroni method, and loci with an adjusted *p* value < 0.01 were considered expressed.

A median value of the intensities of probes was assigned to each locus from the individual array experiments. Probe intensities from each time-series sample (after the perturbation) were compared against those of a reference sample (before the perturbation). A median value of the log_2_-transformed intensity ratios of probes was assigned to each locus. The processed array data were plotted against coordinates on the genome using the Gaggle Genome Browser^60^. The MultiExperiment Viewer (MeV)^61^ was used for hierarchical clustering.

### Quantitative real-time PCR (qRT-PCR)

The gene expression levels of selected genes were further measured with qRT-PCR. Total RNA extracted from the time-series samples was reverse transcribed to cDNA using SuperScript II RTase (Invitrogen, Carlsbad, CA, USA) according to the manufacturer’s instructions. cDNA of the mRNA was amplified using primer pairs (Supplementary Table S5). qRT-PCR was performed using the StepOnePlus Real-Time PCR System (Applied Biosystems, Foster City, CA, USA) using SYBR Green PCR Kit (Applied Biosystems) according to the manufacturer’s instructions. Thermal cycling conditions were 95 °C for 10 min, followed by 40 cycles of 95 °C for 15 s, 62 °C for 15 s, and 15 s at the optimal melting temperature of 72 °C. The data were analyzed using StepOne software v2.2.2 (Applied Biosystems). The expression level of each mRNA was normalized to an endogenous control 16S rRNA and calculated using the 2^−ΔΔCt^ method^62^.

### Metabolome analysis

Intracellular metabolites were measured by GC‐TOF‐MS analysis^63^. Cells were harvested by centrifugation at 13,000 rpm for 3 min. For metabolite extraction, the cell pellet was homogenized with 900 μL of methanol and 10 μL of internal standard (0.5 mg/mL, 2-chlorophenylalanine) and subjected to three freeze–thaw cycles (liquid nitrogen-on ice, each step for 5 min). The extracts were then mixed using an MM400 Mixer Mill (Retsch, Haan, Germany) at a frequency of 30 s^−1^ for 5 min with zirconium beads and sonication for 5 min. After centrifugation (12,578 g for 10 min at 4 °C), the supernatant was filtered through a 0.2-μm polytetrafluoroethylene filter and evaporated using a Modulspin 31 speed vacuum concentrator set at 30 °C (Biotron, Seoul, Korea). The extracts were analyzed by GC–MS after methoximation/silylation^63^. GC–TOF–MS analysis was performed on the 7890 gas chromatograph system (Agilent Technologies) combined with a model 7693 autosampler (Agilent Technologies) and equipped with Pegasus HT TOF MS (LECO, St. Joseph, MI, USA) system as previously described^63^. Helium carrier gas flowed constantly at a rate of 1.5 mL/min through a Rtx-5MS column (30 m × 0.25 mm i.d.; 0.25 µm particle size; Restek Corp., Bellefonte, PA, USA). One microliter of the derivatized samples was injected into the GC–TOF–MS instrument with split ratio of 5:1 in the split 1 mode. The oven temperature was maintained at 75 °C for 2 min, raised to 300 °C at a rate of 15 °C /min, and then held for 3 min. The temperatures of the front inlet and transfer line were set to 250 °C and 240 °C, respectively. The electron ionization was performed at ‒ 70 eV, and the mass spectrometric data were collected in full scanning over a range of 50–1000 m/z.

GC‐TOF‐MS raw data was converted to the NetCDF format (*.cdf) using LECO Chroma TOF software (version 4.44, LECO Corp.). After raw file conversion, peak alignment was processed using MetAlign software (version 041012, https://www.metalign.nl). Metabolomics data was normalized using an internal standard. Multivariate statistical analysis of PCA and PLS-DA was performed using SIMCA P+ software (version 12.0, Umetrics, Umea, Sweden).

### Analysis of intracellular reactive oxygen species (ROS)

The intracellular levels of ROS were measured by staining the cells with dihydroethidium (DHE) redox-sensitive dye to detect the intracellular superoxide radicals. Cell cultures were centrifuged at 13,000 rpm for 3 min. After washing with phosphate-buffered saline (PBS, pH 7.4) and centrifuging twice as described above, the cell pellets were resuspended in PBS buffer (10^6^ cells/mL) and centrifuged. The resulting cell pellets were resuspended in 400 μL of DHE solution (10 μg/mL; Invitrogen) as a fluorescent probe for the detection of generated ROS and were incubated for 1 h in the dark. The samples were evaluated using Gemini Fluorescence Microplate Readers (Molecular Devices, Sunnyvale, CA, USA). Fluorescence values were normalized to the cell density (OD_600_ of 1.0). Cells treated with 2 mM hydrogen peroxide and stained were used as a positive control, and those without treatment and staining were used as a negative control.

### Flow cytometry analysis of cell size

Cell cultures were centrifuged at 13,000 rpm for 3 min. After washing with PBS and centrifuging twice as just described, the cell pellets were resuspended in PBS (10^6^ cells/mL). The resuspended cells were analyzed using a FACSCalibur device (Becton Dickinson & Co., Mountain View, CA, USA). Forward scatter (FSC) and side scatter (SSC) were set in E2 (voltage) and 860 v, respectively. The total single-cell population was gated by plotting FSC versus SSC using a 488 nm laser. Approximately 10,000 events (or cells) were recorded per sample. The test was performed for two biological replicates per time point. Cell size was calculated using the FSC value, basically reflecting the length of rod-shaped bacteria^29^.

### Scanning electron microscopy (SEM)

SEM images were taken at the Korea Research Institute of Bioscience and Biotechnology. Cells were fixed in 2.5% paraformaldehyde-glutaraldehyde solution (pH 7.2) for 2 h without centrifugation. After fixation, the samples were washed with 0.1 M phosphate buffer (pH 7.2) and fixed again with 1% osmium tetroxide in 0.1 M phosphate buffer (pH 7.2) at 25 °C for 2 h. The samples were then dehydrated with a graded ethanol series, and the ethanol was substituted with isoamyl acetate. Finally, after drying in CO_2_, the samples were sputtered with gold in a model SC502 sputter coater (Polaron, Kent, UK) and observed using Quanta 250 FEG SEM (FEI Company, Hillsboro, OR).

### Construction of gene deletion and overexpression strains

In-frame deletion of ECD_02813 was performed using the lambda red recombinase system^64^. The Km^r^ cassette was amplified from plasmid pKD4 using the primer pairs of ECD_02813-Mu-F and ECD_02813-Mu-R (Supplementary Table S5). The resulting PCR products were electroporated into *E. coli* BL21(DE3) carrying plasmid pKD46. The gene deletion mutant was verified by PCR and DNA sequencing, and the inserted Km^r^ cassette was removed using plasmid pCP20. In-frame deletion mutants of *pqqL*, *spy*, and *chiP* were obtained from the Keio collection of single-gene deletion mutants of *E. coli* K-12 BW25113^65^.

Four segments of the capsule gene cluster (ECD_02813 to 02819), including promoters, were amplified by PCR using the genomic DNA of *E. coli* BL21(DE3) as a template, CloneAmp HiFi PCR Premix (Clontech, Mountain View, CA, USA), and the primer pairs (Cap1-IF/Cap1-IR, Cap2-F/Cap2-R, Cap3-F/Cap3-R, and Cap4-F/Cap4-IR overlapping homologous DNA at the ends) (Supplementary Table S5). Each amplified DNA segment was purified and sub-cloned into the T-blunt vector. Plasmid pACYC-Duet carrying the *chloramphenicol acetyltransferase* (*cat*) gene was linearized by inverse PCR using CloneAmp HiFi PCR Premix (Clontech) and the primer pair of Duet-IF/Duet-IR including the homologous sequence of the capsule gene. The four segments were amplified and fused to generate the entire gene cluster, which was ligated into the linearized vector using the EZ-Fusion cloning kit (Enzynomics, Daejeon, Korea) according to the manufacturer’s instructions. The construct was verified by DNA sequencing. The constructed plasmid was electroporated into *E. coli* BL21(DE3).

Growth experiments were performed in 96-well plates using an Epoch 2 Microplate Spectrophotometer (BioTek, Winooski, VT, USA) with shaking (282 cpm double orbital) for 24 h at 37 °C and 44 °C. A seed culture was grown in LB medium at 37 °C, and 1 μL was transferred to 99 μL of fresh MR medium contained in each well. The MR medium was supplemented with 3 g/L glucose (for the gene deletion mutants) and 3 g/L glucose, 0.1 mM isopropyl β-d-1-thiogalactopyranoside, and 25 μg/mL chloramphenicol (for the ΔECD_02813 harboring the pACYC-Duet). OD_600_ was measured every 10 min.

## Supplementary information

Supplementary Table 1.

Supplementary Table 2.

Supplementary Table 3.

Supplementary Information.

## Data Availability

Tiling array data were deposited in the Gene Expression Omnibus database under entry GSE148034. All relevant data are within the paper and its Supplementary Information files.
