# Supplementary material for: Heat-responsive and time-resolved transcriptome and metabolome analyses of Escherichia coli uncover thermo-tolerant mechanisms
Source: Sci Rep. 2020 Oct 19;10:17715. doi: 10.1038/s41598-020-74606-8 (PMC7572479; doi:10.1038/s41598-020-74606-8)
Supplement: Supplementary file 4 — Supplementary Information. [file 41598_2020_74606_MOESM4_ESM.docx]

**SUPPLEMENTARY INFORMATION**

**Heat-responsive and time-resolved transcriptome and metabolome analyses of *Escherichia coli* uncover thermo-tolerant mechanisms**

Sinyeon Kim^1^, Youngshin Kim^1^, Dong Ho Suh^1^, Choong Hwan Lee^1^, Seung Min Yoo^2^, Sang Yup Lee^3^ & Sung Ho Yoon^1,*^

^1^ Department of Bioscience and Biotechnology, Konkuk University, Seoul 05029, Republic of Korea

^2^ School of Integrative Engineering, Chung-Ang University, Seoul 06974, Republic of Korea

^3^ Metabolic and Biomolecular Engineering National Research Laboratory, Department of Chemical and Biomolecular Engineering (BK21 Plus Program), BioProcess Engineering Research Center, Center for Systems and Synthetic Biotechnology, and Institute for the BioCentury, KAIST, Daejeon 34141, Republic of Korea

* Corresponding. syoon@konkuk.ac.kr

**Supplementary Text**

**Genes differentially expressed at the early, middle, and late (C)** **periods of the upshifted temperature.**

To identify significantly overrepresented biological processes in the different time stages (FDR < 0.05), the genes belonging to each time stage from the TTCA analysis (Supplementary Table S2) were subjected to gene ontology (GO) enrichment analysis using the PANTHER (Protein Analysis Through Evolutionary Relationships, http://pantherdb.org) database^1^ (Table 1). For the functional enrichment analysis, 3,413 loci that were expressed in at least one of the nine time-series array data (*p*-value < 0.01) (Supplementary Table S1) were used as a background gene set.

Significantly high expression at all time points under the heat stress was evident in genes encoding outer membrane protein X (*ompX*), L-alanine exporter (*alaE*), and three function unknown proteins (*ybfA*, *ypdK*, and *yjbT*). In contrast to these five genes, many more (n=39) were downregulated throughout the perturbation. The gene set was enriched with those related to flagellar biosynthesis (*flgAMN*, *flgD*, *flgGJK*, and *flhBAE*; false discovery rate [FDR] = 1.89E-09) (Supplementary Fig. S4A) and disaccharide transport (*malEF*, *malK*-*lamB*, and *treB*; FDR = 1.77E-04).

Early-responsive genes included 20 genes whose transcription peaked within 10 min after the temperature upshift and then decreased to a steady-state level (Fig. 2C). Their functions were mostly associated with the well-known HSPs (FDR = 9.71E-13) of chaperones (*dnaKJ*, *grpE*, *ibpA*, and *groESL*) and proteases (*htpG*, *hspQ*, *clpB*, and *hslUV*) for protein folding and degradation, respectively, in the cytoplasm (Supplementary Fig. S4B). These cytosolic HSP genes reached their maximum transcript levels (4- to 8-fold) within only 10 min and thereafter declined to new steady-state levels with different values. Such transient bursts in gene expression were observed in previous studies using flask batch cultures and were reportedly caused by the translational regulation of *σ*^32^ ^2-6^. A membrane-bound protease gene (*ftsH*) also showed a similar expression pattern (Supplementary Table S1). Other early-responsive genes were *argH* and *argI*, which are involved in the arginine biosynthesis pathway, and *metF*, which encodes 5,10-methylenetetrahydrofolate reductase. The transcript levels of the genes at the end of the culture were approximately two-fold higher than the levels before perturbation. Interestingly, four genes with unknown functions (*ybeD*, *ycjX*, *ycjF*, and *yibI*) were identified as early-responsive genes. These genes displayed sharp and transient expression changes upon temperature enhancement (Supplementary Table S2). Among these, *ybeD*, *ycjX*, and *ycjF* were previously reported to be highly induced in *E. coli* with *σ*^H^ overexpression and suggested to be *σ*^H^ regulon members^7,8^. Genes rapidly downregulated in the initial 10 min were those involved in purine biosynthesis (*purB*, *purD*, *purE*, *purL*, and *purT*) and galactose metabolism (*gatB*, *gatC*, and *gatD*) as well as the heat-inducible sigma factor (*rpoE*) and its regulon members (*rseABC*, *yeaY*^9^, and *micA*^10^).

Genes that were highly induced or repressed during the first 2 h of heatshock were identified as early- and middle-responding genes (36 genes) or middle-responding genes (20 genes) (Fig. 2C and Supplementary Table S2). Upregulated genes were those involved in methionine metabolism (*metA*, *metBL*, *metJ*, *metR*, *metN*, *metK*, and *ybdL*) (Supplementary Fig. S4C) and membrane proteins (*ompA*, *ecnB*, *yneM*, and *ybaY*) as well as repressors of biofilms (*bssR*, *bssS*^11^, and *mcbA*^12^). Downregulated genes were those involved in the biosynthesis of purines and pyrimidines (*purC*, *purH*, *purK*, *purM*, and *pyrD*). Genes highly expressed during the middle and late stages were those associated with the phage shock protein (Psp) response (*pspABCDE* and *pspG*)^13^, extra-cytoplasmic chaperones (*spy*, *htpX*, and *cpxP*), and oxidative stress (*yhaK* and *yhcN*).

Many genes were differentially expressed only during the late stage (Fig. 2C). Upregulated genes were mostly linked to iron metabolism: enterobactin biosynthesis (*entCEB*, *entD*, and *fes*-*entF*; FDR = 4.75E-06) (Supplementary Fig. S4D), iron uptake (*fhuA* and *fhuE*), iron-starvation sigma factor (sigma 19) (*fecI*), regulator of *fec* operon (*fecR*), small RNA (sRNA) involved in iron homeostasis (*ryhB*), and bacterioferritin-associated ferredoxin (*bfd*). A large gene cluster (16.3 kb, ECD_02813 to ECD_02828) for capsular polysaccharide biosynthesis (*kps* genes) was also upregulated (Supplementary Fig. S4D). Genes that were significantly downregulated during the late stage were those associated with aldehyde dehydrogenase activity (*aldB*, *astD*, and *patD*) and nutrient transporters (*cynX*, *ydcV*, *glcA*, *garP*, *yhdW*, *ugpB*, and *malG*).

**Coordinated transcriptional responses mediated by sigma factors**

As global transcriptional regulation is controlled by sigma factors, we inspected the expression trajectories of different species of sigma factors (Supplementary Fig. S5A). The expression profiles from the tiling arrays agreed with the quantitative real-time PCR (qRT-PCR) results of samples obtained from three independent chemostats (Supplementary Fig. S5B). *E. coli* BL21(DE3) possesses six sigma factors (*rpoD*, *rpoE*, *rpoH*, *rpoN*, *rpoS*, and *fecI*) but lacks the flagella-specific *rpoF* sigma factor, which is intact in the *E. coli* K-12 strain^14^. The major heat-inducible sigma gene (*rpoH*) was continuously upregulated, whereas extracytoplasmic function (ECF) sigma genes (*rpoE* and *fecI*)^15^ were transiently repressed. The stationary phase sigma factor (RpoS) is a key mediator of the general stress response and was reported to be induced by various stress conditions, including heatshock^16,17^. However, we observed that the expression level of *rpoS* did not respond appreciably to the temperature upshift, indicating that the heatshock condition in this study was not accompanied by entry into the stationary phase. Similarly, *relA* and *spoT*, which encode key enzymes associated with the stringent response to amino acid starvation, were not upregulated.

The predominant sigma factor *rpoD* showed the impulse-like expression pattern that is typical of HSP genes. This finding is not surprising considering that *rpoD* is a *σ*^H^ regulon member and that *σ*^D^ is inactivated at high temperature owing to aggregation^18^ and the prevention of its association with core RNA polymerase^19^. The latter is supported by the previous observation that, in extracts from *E. coli* shifted from 33°C to 40°C or 45°C, the concentration of E*σ*^D^ holoenzyme decreased, whereas that of free *σ*^H^ increased with increasing temperature^19^. Most of the *σ*^H^ regulon genes showed the impulse-like expression pattern (Supplementary Fig. S4B). Intriguingly, the increased *rpoH* transcription upon heat stress did not decrease, even after the induction phase of the HSR. These findings indicated that *σ*^H^ activity after transcriptional activation is primarily regulated at the translational or post-translational level, rather than the transcriptional level^20^. Additionally, maintaining a high level of *rpoH* mRNA during steady-state conditions might still be required for high temperature growth.

The second heatshock sigma factor gene, *rpoE*, was downregulated within 10 min after the heat stress and gradually returned to the level in unstressed cells (Supplementary Fig. S5). This expression pattern was also observed for *σ*^E^ regulon members and *surA*, which encodes the major chaperone for outer membrane proteins (OMPs)^21^ (Supplementary Table S1). Most *rpoE* regulon members were downregulated upon heat stress. Among the 23 highly conserved *σ*^E^ regulon members^22^ (Supplementary Fig. S6A), nine (*bamD*, *degP*, *fkpA*, *plsB*, *rpoE*, *rseA*, *skp*, *yeaY*, and *ygiM*) were immediately downregulated (< 0.5-fold) within 10 min after the temperature increase, whereas *rpoH* and *ompX* were immediately upregulated (> two-fold). Among 106 *σ*^E^ regulon members reported in RegulonDB^23^ (Supplementary Fig. S6B), 23 genes (*bamD*, *bepA*, *degP*, *dnaE*, *eptB*, *fkpA*, *micA*, *plsB*, *rpoE*, *rseA*, *rseB*, *rseC*, *rybB*, *sgbH*, *skp*, *ydhI*, *ydhJ*, *yeaY*, *yfeK*, *yfeS*, *yfgD*, *ygiM*, *yhjJ*, and *yiaO*) were immediately downregulated (< 0.5-fold) within 10 min after the temperature increase, whereas three genes (*rpoD*, *rpoH*, and *yiiS*) were immediately upregulated (> two-fold).

In contrast to our observations, a previous study using flask cultures reported increased *σ*^E^ activity upon the elevation of temperature from 30 to 43°C, although the activity was transiently decreased for the first 2 min^24^. A possible explanation for this discrepancy might be the consistent slow growth observed in this study. The *E. coli* extracytoplasmic proteome, especially OMPs, is considerably more thermostable than the cytoplasmic proteome^25^. Thus, it is conceivable that, when slowly dividing cells are exposed to mild heat stress of 42°C, there is little impact on the folded states of pre-existing periplasmic proteins; further, the accumulation of misfolded periplasmic and OMPs might not be severe enough to trigger the *σ*^E^-mediated response.

It is likely that the decreased amount of proteins newly translocated to the extracytoplasm might be responsible for the downregulation of *rpoE* upon the temperature upshift. Extracytoplasmic proteins are synthesized in the cytoplasm, and they need to pass through the Sec translocon (consisting of SecA and two integral membrane domains, SecYEG and YajC-SecDF) to form a hydrophilic pore in the cytoplasmic membrane^26^. We observed that the *secA* and *yajC-secDF* operon genes were rapidly downregulated (by approximately 0.6-fold) within 10 min after the temperature upshift and thereafter increased up to the levels that existed before perturbation. This pattern was also observed for *secB*, which encodes the cytosolic chaperone that is specifically involved in protein export. In addition, phage shock protein genes (*pspABCDE* and *pspG*) were highly expressed (2- to 4-fold) throughout the temperature upshift. Mutations in *secD*, *secF*, and *yidC* reportedly resulted in the overexpression of the PspA protein, and the *psp* genes were supposedly induced by the slowed Sec-mediated translocation^27^. As the folded state of the cytoplasmic proteins became stabilized upon acclimation to the continued heat stress, the expression levels of the Sec translocon genes were restored to their levels before perturbation, leading to the restoration of *rpoE* expression to the unstressed level.

**Supplementary Figures**

**Figure S1.** **Functional enrichment of differentially expressed genes (DEGs) in response to heatshock stress.** (**A**) Compared to the transcript levels at steady-state culture conditions at 37°C, those that were up or downregulated according to the time-series under heat stress were categorized by clusters of orthologous groups (COGs). (**B**) Comparison of COGs according to DEGs identified in the early (2 and 10 min), middle (0.5, 1, and 2 h), and late (4, 8, and 40 h) stages.

**Figure S2. Clustering of the time-series samples.** (**A**) Hierarchical clustering. The images were created with MeV software (version 4.9.0, http://mev.tm4.org). (**B**) Principal component analysis (PCA). The input data were the expression profiles of the 3,413 genes of which mRNA changes at eight time points at 42°C were calculated in reference to a RNA sample from steady-state growth culture at 37°C. The quality of the PCA model with two components was represented by R2X (cumulative) of 0.692 and Q2 (cumulative) of 0.339. The high values of R2X and Q2 estimate the goodness of fit and predictive ability of the PCA model, respectively. The time-series samples were clearly grouped into three stages of early (2 and 10 min), middle (0.5, 1, and 2 h), and late (4, 8, and 40 h).

**
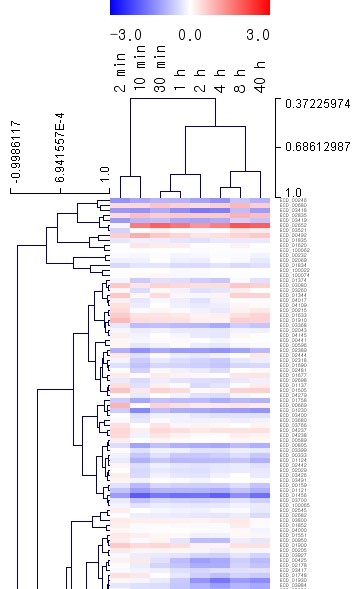

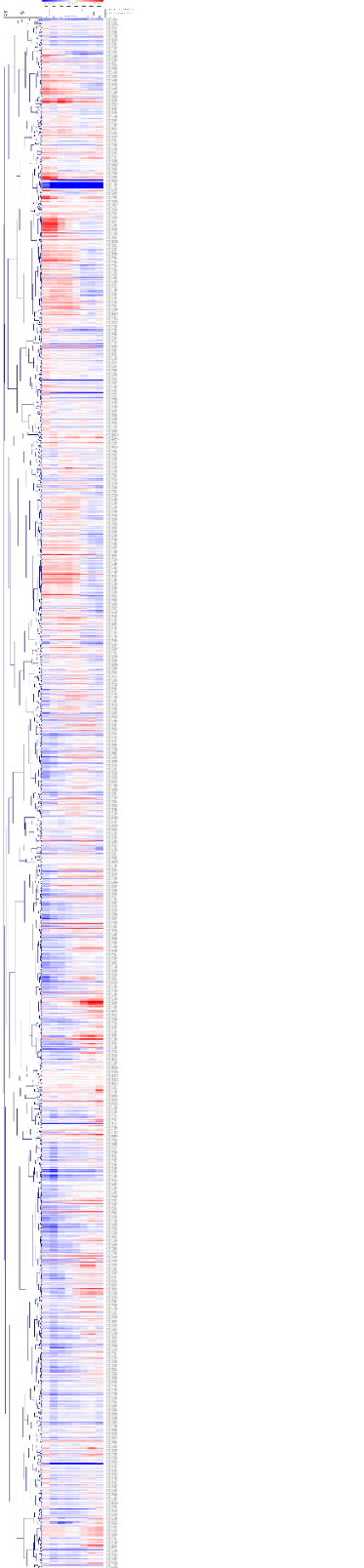
A**

**
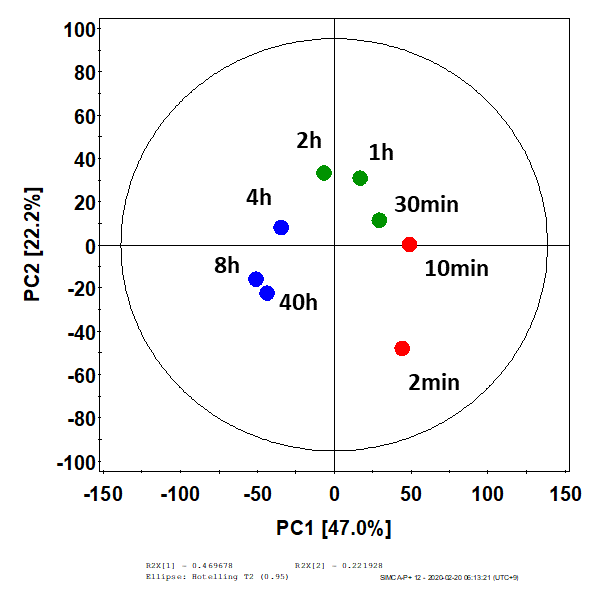
**

**B**

**Figure S3. Identification of genes showing significant dynamics at the early (A), middle (B), and late (C) periods of the upshifted temperature.** Three separate integral scores^28^ of the early, middle, and late stages were computed for 3,413 loci that were expressed in at least one of the nine time-series array data. Then, genes showing significant dynamics (*p*-value < 0.05) were identified from the log-normal distribution function providing the best fit of the distribution of the integral score values in each time interval. The images were created with TTCA^28^ R package.

**A B**


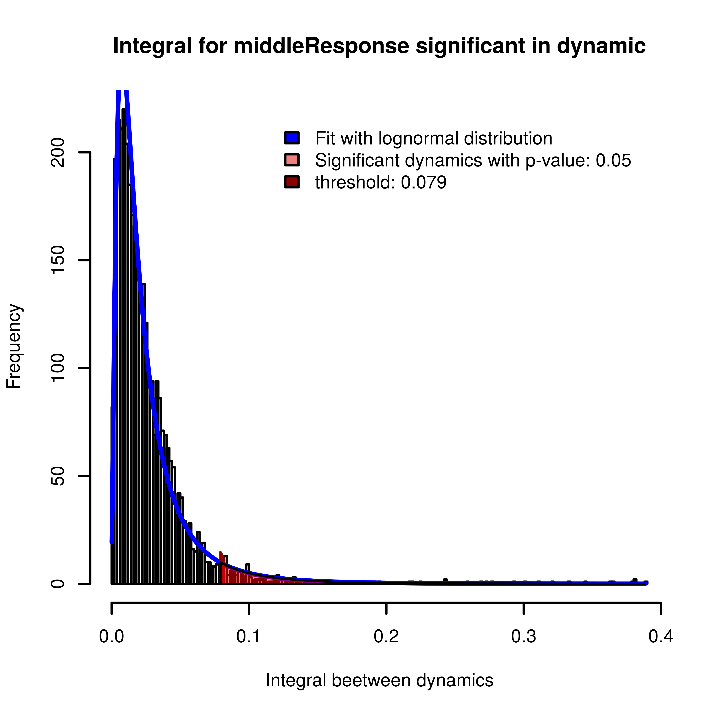

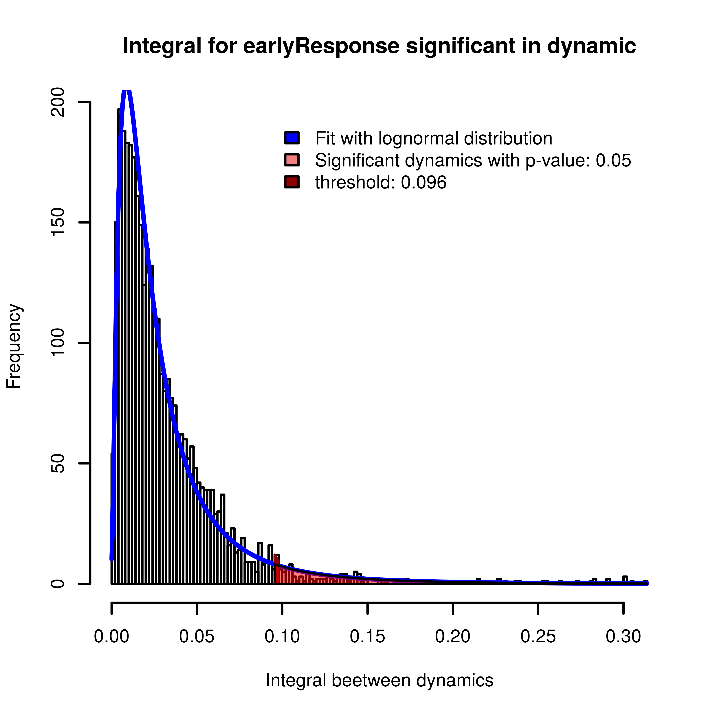


**C**


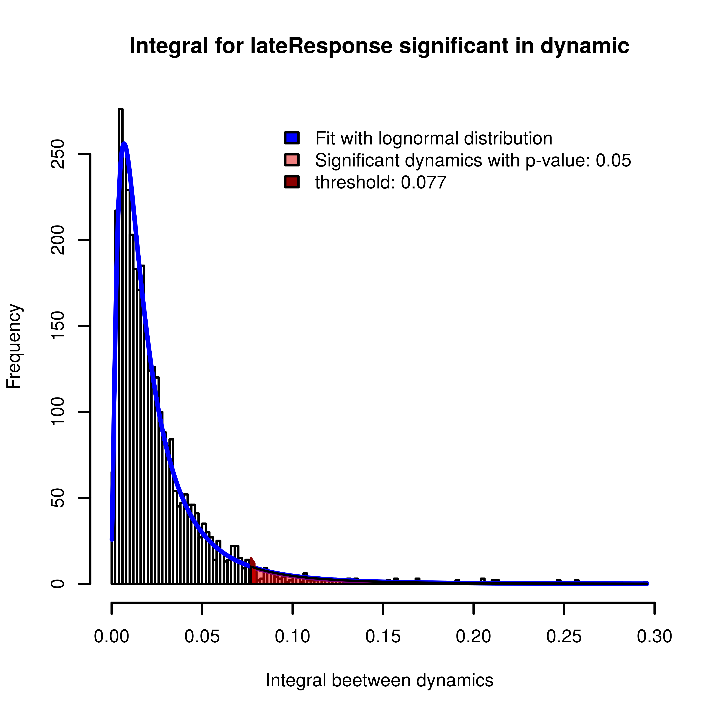


**Figure S4. Temporal dynamics of differentially expressed genes according to early, middle, and late stages.** The tiling array data were plotted against coordinates on the genome using Gaggle Genome Browser^29^. Genes in the forward and reverse strands are represented in yellow and orange, respectively. Corresponding transcriptome data are aligned above the forward strand and below the reverse strand. Dots represent the normalized probe intensities on a log2 scale at the corresponding genomic location for a reference sample grown at 37°C (●) and eight time-series samples at 42°C: 2 min (●), 10 min (●), 30 min (●), 1 h (●), 2 h (●), 4 h (●), 8 h (●), and 40 h (●) [early (2 and 10 min), middle (0.5, 1, and 2 h), and late (4, 8, and 40 h) stages]. Heat maps denote the transcript level changes (log2 scale) of eight time-series samples against the reference sample (green is downregulated and red is upregulated).

**Figure S5. Time profiles of the expression levels of sigma factors observed from tiling arrays (A) and qRT-PCR (B).** The X-axis denotes the culture time after the temperature upshift (min in log scale). The Y axis denotes the transcript ratio in reference to the mRNA intensity 30 min before the perturbation. The error bars represent the standard deviation of the mean from three independent cultivations.

**A**

**B**

**Figure S6. Hierarchical clustering of *rpoE* regulon members.** (**A**) Heatmap of the highly conserved *σ*^E^ regulon members (23 ea) reported in the reference^22^. (**B**) Heatmap of *σ*^E^ regulon members (106 ea) reported in RegulonDB^23^. The mRNA log_2_ ratios of time-series data were normalized to a sample from pre-perturbation (30 min before the temperature upshift). The images were created with MeV software (version 4.9.0, http://mev.tm4.org).


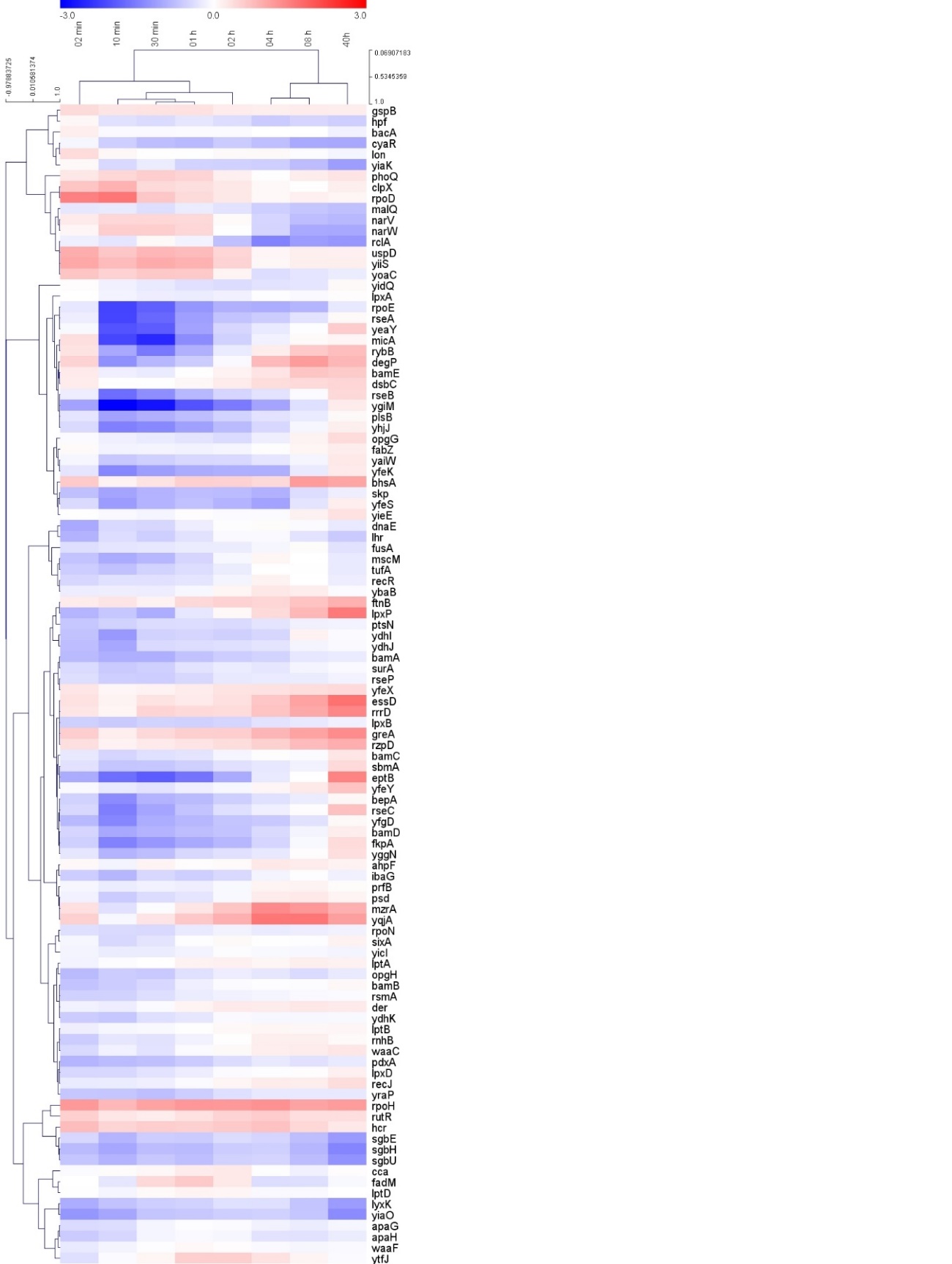

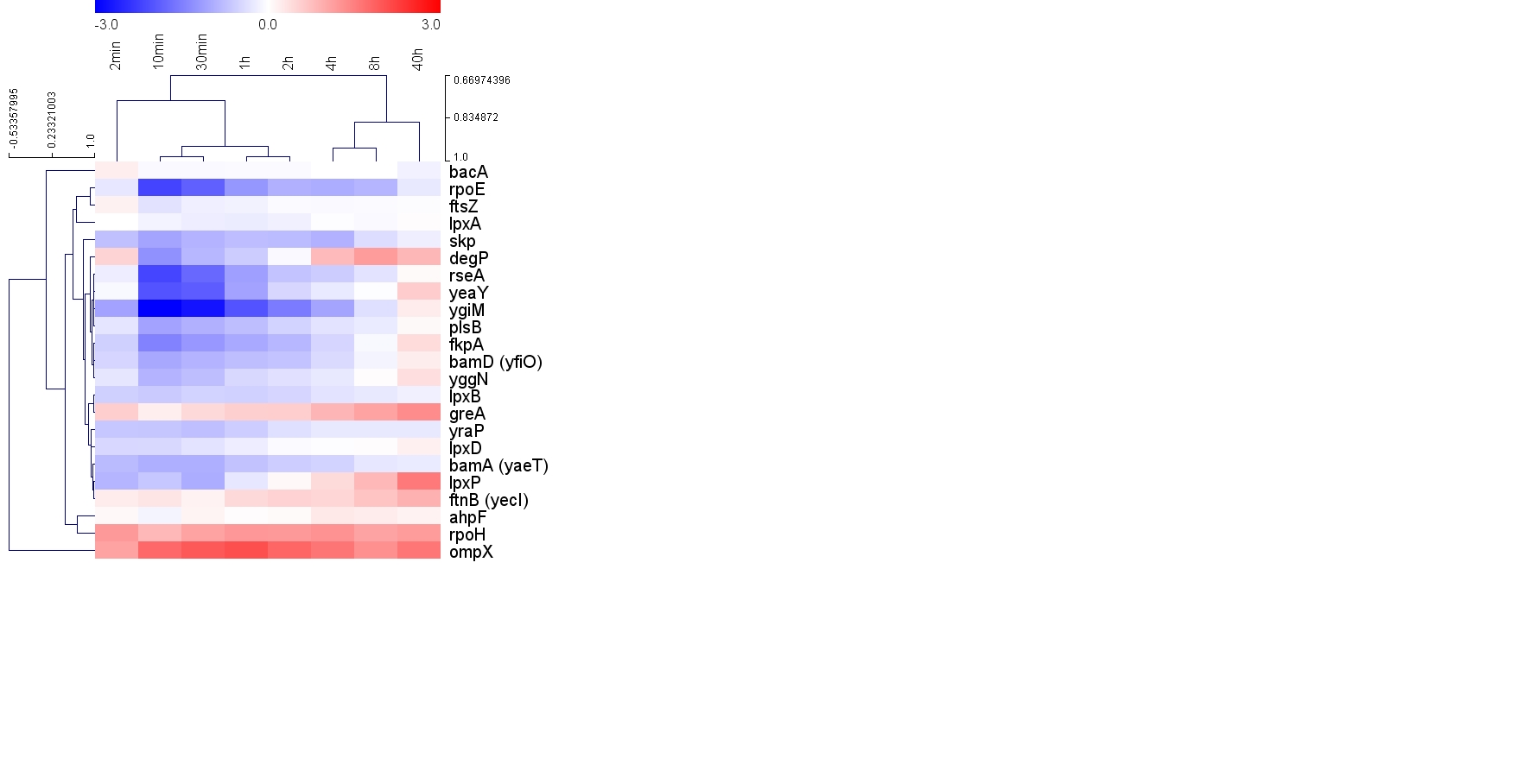
**A B**

**Figure S7. Score plots of PCA and PLS-DA based on the metabolite profiles.** (**A**) The PCA score plot was derived from GC-TOF-MS analysis. (**B**) The PLS-DA score plot was derived from the value of the PCA model. Time-series samples taken after the temperature upshift from 37°C (-30 min; ●) to 42°C were clearly grouped into three stages: early (2 and 10 min; ●), middle (0.5, 1, and 2 h; ●), and late (4, 8, and 40 h; ●) stages. GC-TOF-MS analysis was performed in triplicate for each time point. The quality of the PCA model with two components was estimated by R2X (cumulative) of 0.346 and Q2 (cumulative) of 0.515. These variables were applied to the PLS-DA score plots to identify discriminable variables between the experimental groups based on a model with R2X and R2Y values and Q2. The images were created with SIMCA P+ software (version 12.0, Umetrics, Umea, Sweden).

**Figure S8. Cell size in the time-series samples from the chemostat culture shifted from 37****°C to 42°C.** The percentage of cell size in the total cells was monitored by forward scatter value (FSC) analysis with flow cytometry. The mean FSC-H values corresponding to each histogram curve represent the cell size. The vertical red line denotes the longest 1% size of the -30 min sample (sampling time point 30 min before heatshock). This line was used as the threshold.


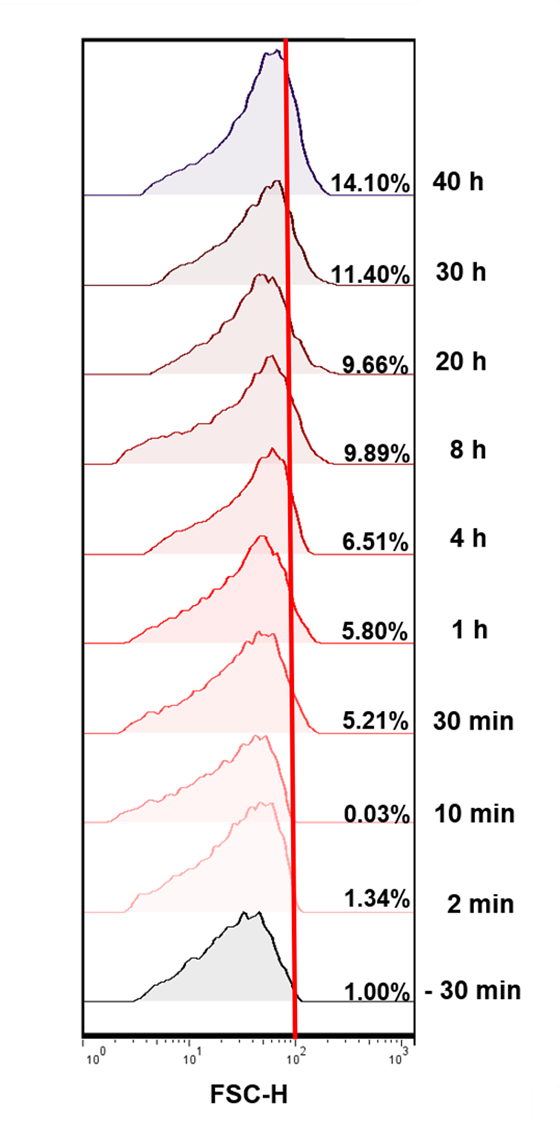


**Figure S9.** **Scanning electron micrographs of the experimental evolution of *E. coli* cells showing morphological plasticity.** The chemostat cultures sampled 40 h after the temperature upshift (42°C) were propagated at 37°C (**A**) or 42°C (**B**) in MR medium by transferring 0.25 mL of the culture into 25 mL of fresh medium every 12 h. After 16 serial transfers (equivalent to about 100 generations), the granule-forming and elongated cells cultured at 42°C for 40 h returned to the regular rod-like form.

**Figure S10.** **Effect of deletion of selected heatshock-responsive genes on bacterial growth at 30**°**C, 37**°**C, and 44**°**C.** (**A**) Growth curves of gene deletion mutants. The *spy* (■), *chiP* (■)*,* and *pqqL* (■) genes were deleted in *E. coli* K-12 BW25113 (□), and ECD_02813 (●) was deleted in *E. coli* BL21(DE3) (●). (**B**) Complementation of ECD_02813 deletion mutant (ΔECD_02813) with the expression of the gene cluster of ECD_02813-02819. The growth of ΔECD_02813 transformed with the empty plasmid (pACYCDuet) (●) was compared with that ΔECD_02813 transformed with pACYCDuet-ECD_02813-02819 (●). BL21(DE3) with the empty plasmid (pACYCDuet) (●) and pACYCDuet-ECD_02813-02819 (●) were included as additional positive controls. The error bars represent the standard deviation of the mean from three independent cultivations.

**Figure S11. *E. coli* metabolic gene and metabolite expression after temperature upshift (37°C to 42°C).** Compared to the sample grown at 37**°**C, the genes and metabolites showing differences in the mRNA and metabolite levels of ≥1.4- or ≤0.7-fold are colored red or blue, respectively, at 10 min (**A**) and 40 h (**B**). Otherwise, they are colored black. Metabolites (in capital letters) identified in this study are highlighted in yellow background. Metabolite abbreviations are 3PG (3-phospho-D-glycerate), 4ABUT (4-aminobutanoate), ACCOA (acetyl-CoA), AKG (2-oxoglutarate), AMET (S-adenosyl-L-methionine), ARG (arginine), ASP (aspartate), CAD (cadaverine), CHOR (chorismate), CIT (citrate), CYS (cystein), RU5P (D-ribulose 5-phosphate), E4P (D-erythrose-4-phosphate), ENTER (enterobactin), F6P (fructose-6-phosphate), FUM (fumarate), G6P (glucose-6-phosphate), GLC (glucose), GLU (glutamate), GXL (glyoxal), HCYS (homocysteine), HIS (histidine), ICIT (isocitrate), D_LAC (D-lactate), L_LAC (L-lactate), LYS (L-lysine), MAL (maltate), MET (methionine), MTHGXL (methylglyoxal), OA (oxaloacetate), PEP (phosphoenolpyruvate), PYR (pyruvate), R5P (alpha-D-ribose 5-phosphate), S7P sedoheptulose 7-phosphate), HSER (homoserine), SUCC (succinate), SUCCOA (succinyl-CoA), TRE (trehalose), TRE6P (trehalose-6-phosphate), and XU5P (xylulose-5-phosphate).

**Supplementary Tables**

**Table S4. *E. coli* strains and plasmids used in this study**

| **Strain or plasmid** | **Description** | **Source or reference** |
| --- | --- | --- |
| **Strains** |  |  |
| BL21(DE3) | Wild type | Lab stock |
| BL21(DE3)ΔECD_02813 | ECD_02813 null mutant of BL21(DE3) | This study |
| BW25113 | Wild type | Keio collection |
| JW0667 | BW25113 *ΔchiP*::Km^r^ | Keio collection |
| JW1489 | BW25113 *ΔpqqL*::Km^r^ | Keio collection |
| JW1732 | BW25113 *Δspy*::Km^r^ | Keio collection |
|  |  |  |
| **Plasmids** |  |  |
| pACYCDuet | Double T7 promoters, P15A ori, low-copy-number cloning vector (Cm^r^) | NEB |
| pACYCDuet-ECD_02813~02819 | pACYCDuet carrying the fragment of wild type ECD_02813~02819 amplified from BL21(DE3) genomic DNA using the primer pairs of Cap1-IF/Cap1-IR, Cap2-F/Cap2-R, Cap3-F/Cap3-R, and Cap4-F/Cap4-IR overlapping homologous DNA at the ends | This study |

**Table S5.** **Primers used for gene deletion mutant construction, cloning, and quantitative real-time PCR (qRT-PCR)**

| **Primer** | **Oligonucleotide sequence (5′→3′)** | **Purpose** |
| --- | --- | --- |
|  |  |  |
| ECD 02813-Mu-F | AATAGTCAAGTAGGAAACATTTTAATAAATGATAAAAATCgtgtaggctggagctgcttc | Construction of ΔECD_02813 |
| ECD 02813-Mu-R | CAGTCACAACTAACCCGAACCCACTATAATTTATTTATGCcatatgaatatcctccttag |  |
| Duet IF | tcaagtatgaCGATCGCTGACGTCGGTACCCTC | Cloning of ECD_02813-02819 |
| Duet IR  Cap1-IF  Cap1-R  Cap2-F  Cap2-R  Cap3-F  Cap3-R  Cap4-F  Cap4-IR | tattaataaCCGATATCCAATTGAGATCTGCC  gatatcggTTATTAATAACATAGTCATACGTTCTT  TCCGTTTCTGCAAAGGCAAGCT  GCCTTTGCAGAAACGGAGATGG  ATCCTCGGTGGTCATACCCTTC  GTATGACCACCGAGGATTACATC  TTCCATTCGCCATAACGGCAGT  CGTTATGGCGAATGGAAGATGGT  gcgatcgTCATACTTGACGCGACAAGCTCT |  |
| rpoD-F | TGAAGCGAACTTACGTCTGG | qRT-PCR |
| rpoD-R | AGAACTTGTAACCACGGCG |  |
| rpoE-F | CCAGAAGGGAGATCAGAAAGC |  |
| rpoE-R | TACCACATCGGGAACATCAC |  |
| rpoH-F | TCGTAATTATGCGGGCTATGG |  |
| rpoH-R | CAGTGAACGGCGAAGGAG |  |
| rpoN-F | CACTACGCCTCGATGTGCAA |  |
| rpoN-R | GCGTATCGTTACGGCTTTCC |  |
| rpoS-F  rpoS-F  16s rRNA-F  16s rRNA-R | TCTCAACATACGCAACCTGG  AGCTTATGGGACAACTCACG  GTCCACGCCGTAAACGATGT  TTAACCTTGCGGCCGTACTC | Normalization control for qRT-PCR |

**Supplementary References**

1 Mi, H., Muruganujan, A., Ebert, D., Huang, X. & Thomas, P. D. PANTHER version 14: more genomes, a new PANTHER GO-slim and improvements in enrichment analysis tools. *Nucleic Acids Res.* **47**, D419-d426 (2019).

2 Zhao, K., Liu, M. & Burgess, R. R. The global transcriptional response of *Escherichia coli* to induced *σ*^32^ protein involves *σ*^32^ regulon activation followed by inactivation and degradation of *σ*^32^ in vivo. *J. Biol. Chem.* **280**, 17758-17768 (2005).

3 Jozefczuk, S. *et al.* Metabolomic and transcriptomic stress response of *Escherichia coli*. *Mol. Syst. Biol.* **6**, 364 (2010).

4 Gasch, A. P. *et al.* Genomic expression programs in the response of yeast cells to environmental changes. *Mol. Biol. Cell* **11**, 4241-4257 (2000).

5 Straus, D. B., Walter, W. A. & Gross, C. A. The heat shock response of *E. coli* is regulated by changes in the concentration of sigma 32. *Nature* **329**, 348-351 (1987).

6 Richter, K., Haslbeck, M. & Buchner, J. The heat shock response: life on the verge of death. *Mol. Cell.* **40**, 253-266 (2010).

7 Nonaka, G., Blankschien, M., Herman, C., Gross, C. A. & Rhodius, V. A. Regulon and promoter analysis of the *E. coli* heat-shock factor, σ^32^, reveals a multifaceted cellular response to heat stress. *Genes Dev.* **20**, 1776-1789 (2006).

8 Kim, S., Kim, Y. & Yoon, S. H. Overexpression of YbeD in *Escherichia coli* enhances thermotolerance. *J. Microbiol. Biotechnol.* **29**, 401-409 (2019).

9 Rezuchova, B., Miticka, H., Homerova, D., Roberts, M. & Kormanec, J. New members of the *Escherichia coli* σ^E^ regulon identified by a two-plasmid system. *FEMS Microbiol. Lett.* **225**, 1-7 (2003).

10 Johansen, J., Rasmussen, A. A., Overgaard, M. & Valentin-Hansen, P. Conserved small non-coding RNAs that belong to the σ^E^ regulon: role in down-regulation of outer membrane proteins. *J. Mol. Biol.* **364**, 1-8 (2006).

11 Domka, J., Lee, J. & Wood, T. K. YliH (BssR) and YceP (BssS) regulate *Escherichia coli* K-12 biofilm formation by influencing cell signaling. *Appl. Environ. Microbiol.* **72**, 2449-2459 (2006).

12 Zhang, X. S., Garcia-Contreras, R. & Wood, T. K. *Escherichia coli* transcription factor YncC (McbR) regulates colanic acid and biofilm formation by repressing expression of periplasmic protein YbiM (McbA). *ISME J.* **2**, 615-631 (2008).

13 Darwin, A. J. The phage-shock-protein response. *Mol. Microbiol.* **57**, 621-628 (2005).

14 Jeong, H. *et al.* Genome sequences of *Escherichia coli* B strains REL606 and BL21(DE3). *J. Mol. Biol.* **394**, 644-652 (2009).

15 Brooks, B. E. & Buchanan, S. K. Signaling mechanisms for activation of extracytoplasmic function (ECF) sigma factors. *Biochim. Biophys. Acta* **1778**, 1930-1945 (2008).

16 Muffler, A., Barth, M., Marschall, C. & Hengge-Aronis, R. Heat shock regulation of σ^S^ turnover: a role for DnaK and relationship between stress responses mediated by σ^S^ and σ^32^ in *Escherichia coli*. *J. Bacteriol.* **179**, 445-452 (1997).

17 Battesti, A., Majdalani, N. & Gottesman, S. The RpoS-mediated general stress response in *Escherichia coli*. *Annu. Rev. Microbiol.* **65**, 189-213 (2011).

18 Blaszczak, A., Zylicz, M., Georgopoulos, C. & Liberek, K. Both ambient temperature and the DnaK chaperone machine modulate the heat shock response in *Escherichia coli* by regulating the switch between *σ*^70^ and *σ*^32^ factors assembled with RNA polymerase. *Embo J.* **14**, 5085-5093 (1995).

19 Skelly, S., Coleman, T., Fu, C. F., Brot, N. & Weissbach, H. Correlation between the 32-kDa σ factor levels and in vitro expression of *Escherichia coli* heat shock genes. *Proc. Natl. Acad. Sci. USA* **84**, 8365-8369 (1987).

20 Gross, C. A. *Function and regulation of the heat shock proteins* in *Escherichia coli and Salmonella* (ed. Neidhardt, F. C.) 1382-1399 (ASM Press, 1996).

21 Plummer, A. M. & Fleming, K. G. From chaperones to the membrane with a BAM! *Trends Biochem. Sci.* **41**, 872-882 (2016).

22 Rhodius, V. A., Suh, W. C., Nonaka, G., West, J. & Gross, C. A. Conserved and variable functions of the σ^E^ stress response in related genomes. *PLoS Biol.* **4**, e2 (2006).

23 Santos-Zavaleta, A. *et al.* RegulonDB v 10.5: tackling challenges to unify classic and high throughput knowledge of gene regulation in *E. coli* K-12. *Nucleic Acids Res.* **47**, D212-d220 (2019).

24 Ades, S. E., Grigorova, I. L. & Gross, C. A. Regulation of the alternative sigma factor σ^E^ during initiation, adaptation, and shutoff of the extracytoplasmic heat shock response in *Escherichia coli*. *J. Bacteriol.* **185**, 2512-2519 (2003).

25 Mateus, A. *et al.* Thermal proteome profiling in bacteria: probing protein state in vivo. *Mol. Syst. Biol.* **14**, e8242 (2018).

26 Manting, E. H. & Driessen, A. J. *Escherichia coli* translocase: the unravelling of a molecular machine. *Mol. Microbiol.* **37**, 226-238 (2000).

27 Jones, S. E., Lloyd, L. J., Tan, K. K. & Buck, M. Secretion defects that activate the phage shock response of *Escherichia coli*. *J. Bacteriol.* **185**, 6707-6711 (2003).

28 Albrecht, M. *et al.* TTCA: an R package for the identification of differentially expressed genes in time course microarray data. *BMC Bioinformatics* **18**, 33 (2017).

29 Bare, J. C., Koide, T., Reiss, D. J., Tenenbaum, D. & Baliga, N. S. Integration and visualization of systems biology data in context of the genome. *BMC Bioinformatics* **11**, 382 (2010).
